# Comparative analysis of nucleus-encoded plastid-targeting proteins in *Rafflesia cantleyi* against photosynthetic and non-photosynthetic representatives reveals orthologous systems with potentially divergent functions

**DOI:** 10.1038/s41598-018-35173-1

**Published:** 2018-11-22

**Authors:** Siuk-Mun Ng, Xin-Wei Lee, Mohd-Noor Mat-Isa, Mohd Afiq Aizat-Juhari, Jumaat Haji Adam, Rahmah Mohamed, Kiew-Lian Wan, Mohd Firdaus-Raih

**Affiliations:** 1 School of Biosciences and Biotechnology, Faculty of Science and Technology, Universiti Kebangsaan Malaysia, 43600 UKM Bangi, Selangor Malaysia; 2 Malaysia Genome Institute, Jalan Bangi, 43000 Kajang, Selangor Malaysia; 3 School of Environmental and Natural Resource Sciences, Faculty of Science and Technology, Universiti Kebangsaan Malaysia, 43600 UKM Bangi, Selangor Malaysia; 4 Centre for Frontier Sciences, Faculty of Science and Technology and Institute of Systems Biology, Universiti Kebangsaan Malaysia, 43600 UKM Bangi, Selangor Malaysia; 5 Present Address: Codon Genomics SB, No 26, Jalan Dutamas 7, Taman Dutamas Balakong, 43200 Seri Kembangan, Selangor Malaysia

## Abstract

Parasitic plants are known to discard photosynthesis thus leading to the deletion or loss of the plastid genes. Despite plastid genome reduction in non-photosynthetic plants, some nucleus-encoded proteins are transported back to the plastid to carry out specific functions. In this work, we study such proteins in *Rafflesia cantleyi*, a member of the holoparasitic genus well-known for producing the largest single flower in the world. Our analyses of three transcriptome datasets, two holoparasites (*R*. *cantleyi* and *Phelipanche aegyptiaca*) and one photosynthetic plant (*Arabidopsis thaliana*), suggest that holoparasites, such as *R*. *cantleyi*, retain some common plastid associated processes such as biosynthesis of amino acids and lipids, but are missing photosynthesis components that can be extensions of these pathways. The reconstruction of two selected biosynthetic pathways involving plastids correlates the trend of plastid retention to pathway complexity - transcriptome evidence for *R*. *cantleyi* suggests alternate mechanisms in regulating the plastidial heme and terpenoid backbone biosynthesis pathways. The evolution to holoparasitism from autotrophy trends towards devolving the plastid genes to the nuclear genome despite the functional sites remaining in the plastid, or maintaining non-photosynthetic processes in the plastid, before the eventual loss of the plastid and any site dependent functions.

## Introduction

Chloroplasts have long been recognized for their role in supporting plant growth and development^1^. Generally, chloroplast-localized proteins are either responsible for the localization and transport functions within the chloroplast or for chloroplast translation^1^ to carry out biosynthesis of fatty acids, amino acids, vitamins and nucleotides. Plastidial activities have also been shown to be involved in the synthesis of several plant hormones such as abscisic acids that are derived from isoprenoids, gibberrelins and brassinosteroids^2^. Non-photosynthetic parasitic plants source their carbon requirements directly from their respective hosts and the resulting relaxed selective pressures can be seen on their much reduced plastid genome^3^. As holoparasites adopt heterotrophy, many genes required for their original functions are believed to have been transferred to the nucleus^4^ or are lost. This phenomenon is also observed for other plants where the chloroplast genes are transferred to the nuclear genome as a result of evolutionary progression^5^. These genes are encoded in the nucleus, translated in the cytoplasm and their products are then transported back to the plastid^5,6^. The remnant plastid structure provides an overview of how a plastid genome evolves and its residual functions reveal the possible functionality of a reduced plastome. However, a recent study suggests that the chloroplast genome in the genus *Rafflesia* may be absent or present at very low levels, where plastidial fragments are only detected in sizes ranging from 104 to 1,026 bp^7^. Likewise, work on the non-photosynthetic green alga genus, *Polytomella*, revealed that they harbour plastids without a genome^8^.

The habitat of the rafflesiaceae family that the *Rafflesia* genus belongs to are confined to the tropical rainforests of Southeast Asia^9^. The species used in this work, *Rafflesia cantleyi*, is an obligate holoparasite that depends entirely on its host plant, *Tetrastigma rafflesiae*, for all nutrients including inorganic matter such as water^10^. In a comparative study of parasitic plants, *Phelipanche aegyptiaca*, a holoparasite belonging to the Orobanchaceae family was reported to maintain a complete, expressed and partially pressurized chlorophyll synthesis pathway despite its heterotrophic nature^11^. In parasites where the photosynthetic apparatus has been abandoned; the loss of selection pressure on the plastid genome, or more critically on the photosynthesis-related genes has been reported^12^. In reality, plastidial functions rely on both plastid- and nucleus-encoded proteins^13^. The genes involved in the plastid translational apparatus are also confined to both the plastid and the nucleus divisions^14^. Since most plastid genomes are functional, including in holoparasitic plants, nucleus-encoded proteins targeted to the plastid that compensate for gene losses are of great interest in understanding the residual functions of the reduced plastid in holoparasites.

Convolvulaceae and Orobanchaceae are two families that are often used for studying the evolution of holoparasitism from hemiparasitism because they have species ranging from hemi- to holoparasites^15^. We report here the results of a comparative survey of nucleus-encoded proteins targeting the plastid using RNA-Seq data obtained from Lee *et al*.^16^ and public databases. We observed that *R*. *cantleyi* possesses a relatively versatile nucleus-encoded plastid (NEP) RNA polymerase translation that conserves some common plastidial functions while adopting alternative route(s) to compensate for gene loss. The readily accessible bud transcriptome data of a holoparasite, *P*. *aegyptiaca*, along with the photosynthetic model plant *Arabidopsis thaliana* were used for comparison against our *R*. *cantleyi* subject.

## Results

### Genome assembly and annotation of the organellar genes

**Figure 1:**
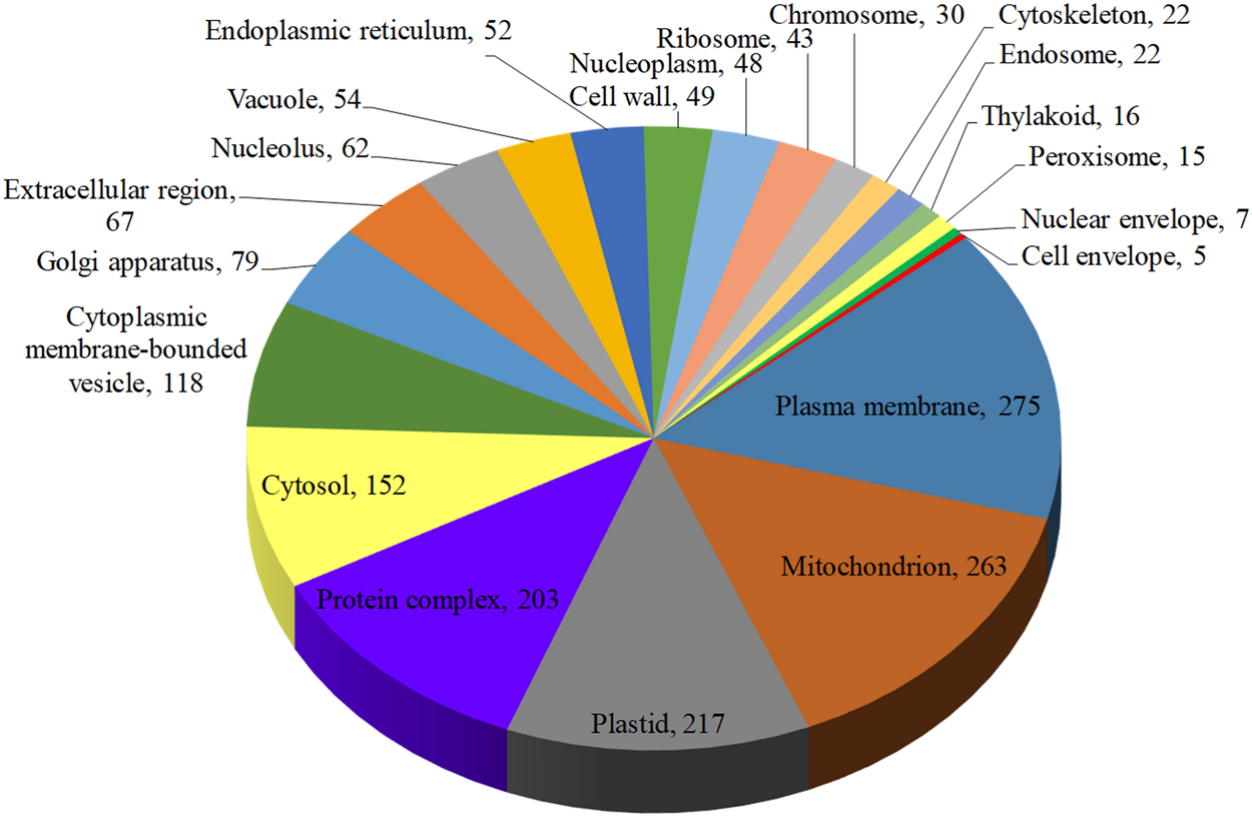
Genome sequence distribution of annotated singletons of *Rafflesia cantleyi* according to cellular location by Blast2GO. A total of 1799 singletons were annotated and categorized according to their cellular location. From that, 217 sequences were found to be plastid-localized, out of which 16 are of thylakoid origin. This chart does not show the abundance of nucleus-encoded plastid-targeting genes, thus implying more genes are involved in plastid metabolic processes.

Roche 454 shotgun sequencing of the *R*. *cantleyi* genome generated a total of 1,091,861 high quality reads with an average length of 289 bp. Following sequence clustering, 607,596 DNA sequences were obtained and assembled into 4,944 contigs and 302,176 singletons. Classification and annotation of the 4,944 contigs using blastx and Blast2GO revealed a prevalence of mitochondrial genes. The singletons were therefore used instead for gene detection. Following BLAST annotation, the singletons were parsed to Blast2GO resulting in 1,799 out of 302,176 singletons being functionally annotated. Of these 1799 functionally annotated singletons, 217 were identified to be of plastid-origin, and 16 sequences were confined to homologs localized in the thylakoid (Fig. 1). Since many, if not most, non-photosynthetic organisms retain a functional plastid despite losing their photosynthetic genes, we focused the subsequent analyses on the nucleus-encoded plastid-targeting proteins commonly known to be present in photosynthetic plants that are responsible for several biosynthetic processes other than photosynthesis^13^. These subsequent analyses were carried out using transcriptome data.

### Identification and functional implication of nucleus-encoded plastid-targeting proteins

**Figure 2:**
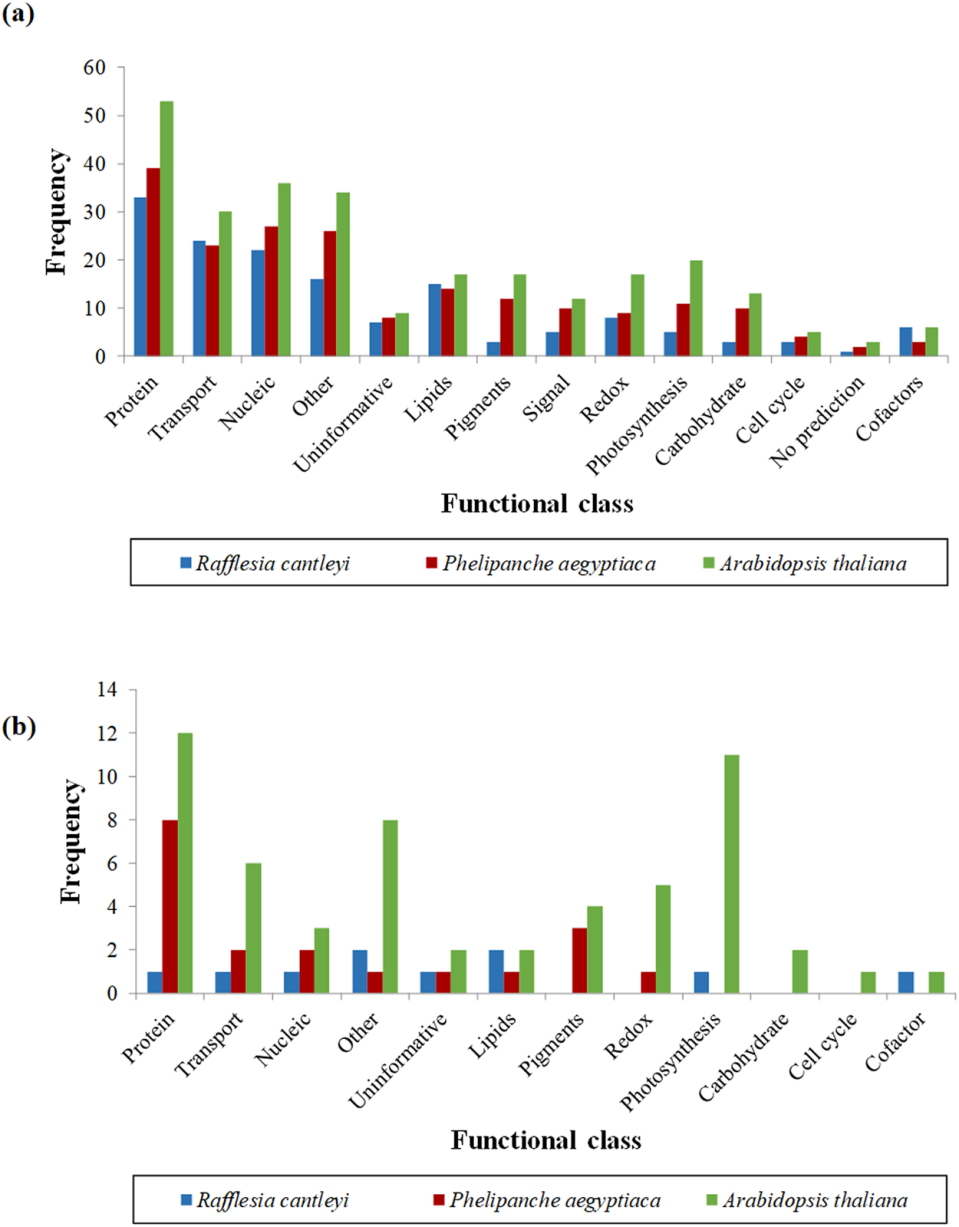
Identification of nucleus-encoded plastid-targeting proteins across *Rafflesia cantleyi*, *Phelipanche aegyptiaca* and *Arabidopsis thaliana* using the criteria described in methods. Putative nucleus-encoded plastid-targeting proteins with a predicted transit peptide (**a**); a predicted transit peptide and are targeted to the chloroplast (**b**) were sorted according to the GreenCut2 functional classification system. Nucleus-encoded plastid-targeting proteins retrieved from both non-photosynthetic holoparasites and photosynthetic green plants are presented.

A total of three transcriptome data sets were compared in this study. The *R*. *cantleyi* transcriptome was sequenced in-house, producing 40,768,187 high quality reads^16^. The consensus contigs of *P*. *aegyptiaca*^17^ and the raw transcriptome data for *A*. *thaliana* were obtained online as described in the methodology section. After transcriptome assembly, the numbers of transcripts produced were 18,053 for *R*. *cantleyi* and 43,783 for *A*. *thaliana*, whereas 68,683 contigs were available for *P*. *aegyptiaca*. Putative nucleus-encoded proteins with the presence of a transit peptide detected for each data set were 150 for *R*. *cantleyi*, 198 for *P*. *aegyptiaca* and 272 for *A*. *thaliana* (Fig. 2a). The full putative protein list is given in the same order as Supplementary Dataset S1a, S1b, S1c. On average, the lengths of predicted transit peptides ranged from 10 to 80 bp. Since each of the transcriptome sizes differed, the lists of putative plastid-localized proteins could perhaps better serve as a reference for future experimental protein characterization rather than as a definitive means of revealing the true functions of the retained plastid of *R*. *cantleyi*. To increase the confidence level that these NEPs were putatively plastid localized, subcellular localization analysis was performed. This resulted in a further reduction of the plastid-localized proteins to 10 for *R*. *cantleyi*, 19 for *P*. *aegyptiaca* and 57 for *A*. *thaliana* (Fig. 2b). The full putative protein list is given in the same order as Supplementary Dataset S2a, S2b, S2c. The predicted nucleus-encoded plastid-targeting proteins in *R*. *cantleyi* and other plants do not represent the complete set of plastid-targeting proteins available for each plant. For this work, we focused on those with relevance to genes contained within the GreenCut2 Resource^13^ along with a predicted chloroplast transit peptide and a predicted localization to the plastid.

Next, the putative plastid-localized proteins were compared based on the MapMan annotation provided in the GreenCut2 inventory. Of the 14 GO annotation groups; “protein”, “transport”, “nucleic” activities and “other” processes that consisted of RNA regulation, fibrillins, amino acid and minor carbohydrate metabolism made up the four dominant functional categories in the three species surveyed. Photosynthetic genes were mostly observed for *A*. *thaliana* and some for *P*. *aegyptiaca*, whereas the *Rafflesia* transcriptome did not show any significant presence of such genes. Functional categories of “carbohydrate”, “cell cycle” and “cofactors” were represented by fewer numbers of nucleus-encoded plastid-targeting proteins, possibly due to the classification parameters where some were instead grouped under “protein” and “other” categories. In this study, genes that were observed to be more commonly expressed than others (Table 1), were considered as components of well-conserved regulatory pathways not only for green photosynthetic plants but also for non-photosynthetic parasites. Some pathways and regulatory steps such as transfer of activated nucleotide sugars to acceptor molecules by glycosyltransferase^18^; modulation of the redox environment via the reduction of disulfide bridges in enzymes by thioredoxin^19^; mediation of TCA, glyoxylate bypass, amino acid synthesis, exchange of metabolites and gluconeogenesis by malate dehydrogenase^20^; and detoxification-associated pathways by glutathione s-transferase^21^ were deemed to remain functional except those specific for food production. In addition, the location of each gene as annotated may be indicative of the cellular location where they carry out their functions.

**Table 1:** Genes that are commonly expressed in the holoparasitic and photosynthetic species analysed in this study.

Annotation	Functional Category	Location	MapMan function category
60S ribosomal protein L10	Protein	S	protein.synthesis.chlorop/mito.plastid
F-box protein	Protein	U	protein.degradation.ubiquitin
calmodulin binding protein-like	Protein	T	signalling.calcium
arginine N-methyltransferase	Protein	Y	methyl transferases
peptidyl-prolyl cis-trans isomerase	Protein	L	protein.folding
SNARE associated Golgi protein family	Transport	C	not assigned.unknown
solute carrier family	Transport	C	not assigned.no ontology
potassium transporter	Transport	C	transport.potassium
ABC transporter family protein	Transport	Y	transport.ABC transporters and multidrug resistance systems
DEAD/DEAH box RNA helicase	Nucleic	C	RNA. DEAD/DEAH BOX helicase
zinc finger family protein	Nucleic	U	protein.degradation.ubiquitin
tRNA/rRNA methyltransferase	Nucleic	Y	not assigned.no ontology
glutathione S-transferase	Redox	T	redox.glutaredoxins
glutaredoxin	Redox	U	transport.calcium
thioredoxin	Redox	C	redox.thioredoxin
malate dehydrogenase	Other	M	TCA/org. transformation.TCA.
amidase	Other	Y	miscellaneous nitrilases
protein phosphatase 2C	Signal	U	protein.postranslational modification
serine/threonine kinases	Signal	C	protein.postranslational modification
glycosyl transferase	Carbohydrate	P	cell wall.cellulose synthesis
dynamin-related protein	Cell cycle	oV	cell.division.plastid
pollen specific protein	Unknown	C	not assigned.unknown

C = chloroplast, L = lumen, M = mitochondrion, P = plasma membrane, S = stroma, T = thylakoid membrane, U = nucleus, oV = outer envelope, Y = cytoplasm.

The transcription status of both NEP and non-NEP related genes were annotated based on their presence in the available transcriptome data. In this study, riboflavin synthase and lysophosphatidic acid acyltransferase, known for participating in riboflavin metabolism, and phosphatidic acid biosynthesis, were investigated using a phylogenetic approach (Supplementary Fig. S1). These genes were chosen due to their importance in providing indispensable precursors or intermediates for the plant’s survival. The shorter distance between *Rafflesia* and its known close relatives suggests that these genes were unlikely acquired from its host through horizontal gene transfer but were transcribed and expressed to fulfill specific biological roles. For instance, riboflavin synthase serves to produce vitamin B2, a precursor that further supplies essential cofactors for several cellular processes such as the citric acid cycle, fatty acid oxidation, and mitochondrial electron transport, therefore showing the importance of riboflavin accessibility^22^.

### Pathway reconstruction in *R*. *cantleyi*

**Figure 3:**
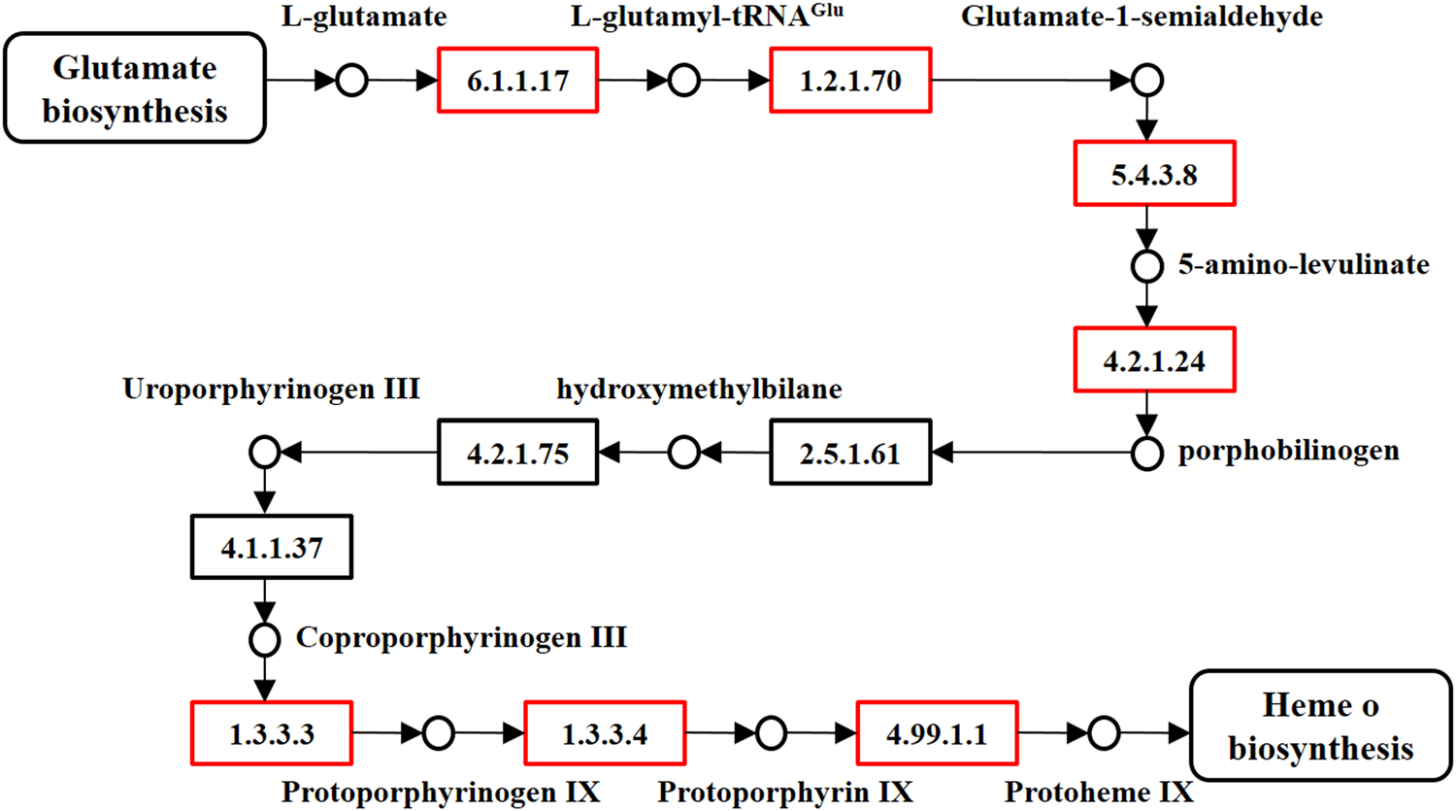
Analysis of heme biosynthesis pathway using genome and transcriptome data. This pathway consists of genes that mapped to that of *Arabidopsis thaliana* col heme biosynthesis I embedded within Plant Metabolic Network database. At the genome level, all ten genes were present whereas only eight genes (labeled with red box) were detected at transcript level for *Rafflesia cantleyi*. Using our assembled *Arabidopsis* transcriptome, all ten genes required in this process were retrieved.

Two pathways were reconstructed to contrast the differences between a photosynthetic and a non-photosynthetic plant. These two pathways were chosen based on their retained presence in both parasitic and non-parasitic plants. The first pathway is the porphyrin biosynthesis pathway. Intermediate products of heme biosynthesis were observed for some genes but this pathway appears to end at heme production in *R*. *cantleyi* (Fig. 3). As expected, chlorophyll synthesis genes were not transcribed in *R*. *cantleyi*. Although identical initial steps for heme production and chlorophyll synthesis were present, the expression of genes directly involved in chlorophyll synthesis were not observed, thus alluding that this process no longer takes place in this holoparasite.

**Figure 4:**
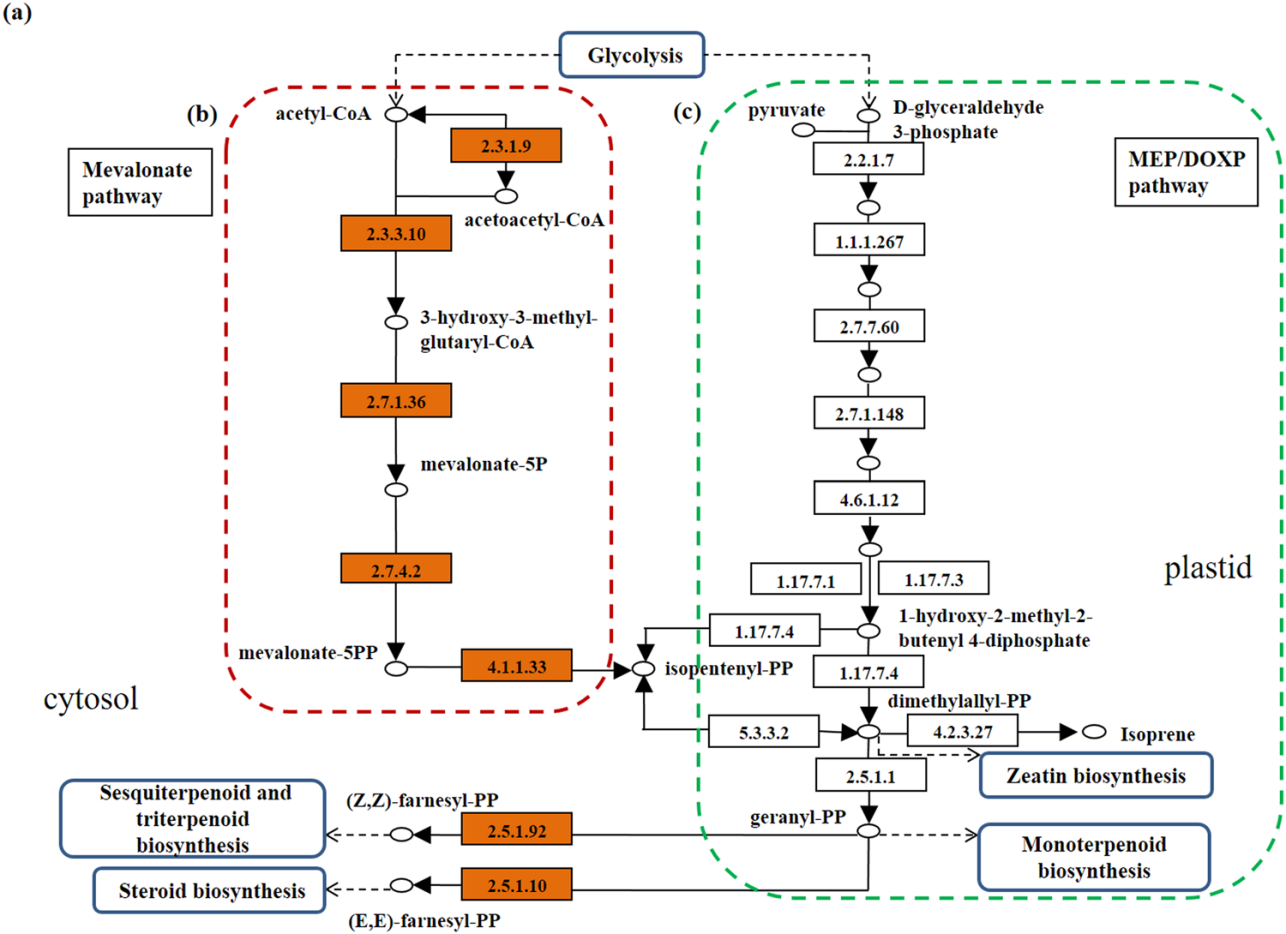
Reconstructed terpenoid backbone biosynthesis for *Rafflesia cantleyi* at transcript level. This pathway was reconstructed based on the detection of transcripts present. Of the (**a**) two mechanisms present for IPP production, *Rafflesia* is found to consist of only the (**b**) genes present in cytoplasm but not (**c**) genes in the plastid. *Rafflesia*-containing enzymes with EC numbers include: 2.3.1.9, acetyl-CoA c-acetyltransferase; 2.3.3.10, hydroxymethylglutaryl-CoA synthase; 2.7.1.36, mevalonate kinase; 2.7.4.2, phosphomevalonate kinase; 4.1.1.33, diphosphomevalonate decarboxylase; 2.5.1.1, 2.5.1.10, farnesyl diphosphate synthase; 2.5.1.92, (2z,6z)-farnesyl diphosphate synthase; whereas enzymes confined to the plastid were labeled with EC numbers: 2.2.1.7, 1-deoxy-D-xylulose-5-phosphate synthase; 1.1.1.267, 1-deoxy-D-xylulose-5-phosphate reductoisomerase; 2.7.7.60, 2-*C-*methyl-D-erythritol 4-phosphate cytidylyltransferase; 2.7.1.148, 4-(cytidine 5′-diphospho)-2-C-methyl-D-erythritol kinase; 4.6.1.12, 2-*C-*methyl-D-erythritol 2,4-cyclodiphosphate synthase; 1.17.7.1, (E)-4-hydroxy-3-methylbut-2-enyl-diphosphate synthase; 1.17.7.3, (*E*)-4-hydroxy-3-methylbut-2-enyl-diphosphate synthase; 1.17.7.4, 4-hydroxy-3-methylbut-2-en-1-yl diphosphate reductase; 5.3.3.2, isopentenyl-diphosphate Δ-isomerase; 4.2.3.27, isoprene synthase.

Reconstruction of the terpenoid backbone biosynthesis pathway (Fig. 4) enabled the detection of genes that are involved in the mevalonate pathway. *R*. *cantleyi* was observed to be similar to other heterotrophic plants where the cytoplasm becomes the site for this biosynthetic process. This suggests that the parasite potentially takes up an alternative path within the cytoplasm due to the reduction of the plastid genome.

### Analysis of *Rafflesia cantleyi* orthologous NEP genes

The ten putative nucleus-encoded plastid-targeting proteins (Supplementary Dataset S2a) of *Rafflesia* were used to search for their orthologs using OrthoMCL^23^. The orthologous groups identified for eight putative proteins are listed in Table 2. The orthologous groups are made up of biosynthetic and metabolic processes, regulation of transcription and response to stress. This characterization according to GO biological processes and molecular function reveals the potentially retained functions in the *Rafflesia* plastid.

**Table 2:** Identification of orthologous groups for *Rafflesia* putative nucleus-encoded plastid-targeting proteins using OrthoMCL.

Orthologous group ID	Gene description	GO molecular function	GO biological processes
1059	TCP-1/cpn60 chaperonin family	ATP binding (GO:0005524)	cellular protein metabolic process (GO:0044267)
3655	aspartate aminotransferase	pyridoxal phosphate binding (GO:0030170)	biosynthetic process (GO:0009058)
1055	Plant stearoyl-acyl-carrier-protein desaturase family protein	acyl-[ACP] desaturase activity (GO:0045300)	fatty acid metabolic process (GO:0006631);
6960	Adenine nucleotide alpha hydrolases-like superfamily protein	—	oxidation-reduction process (GO:0055114) response to stress (GO:0006950)
1965	GATA transcription factor 26	sequence-specific DNA binding (GO:0043565)	regulation of transcription, DNA-templated (GO:0006355)
2181	ARF-GAP domain 9	zinc ion binding (GO:0008270); ARF GTPase activator activity (GO:0008060)	regulation of ARF GTPase activity (GO:0032312)
7333	lumazine-binding family protein	—	—
2050	O-fucosyltransferase family protein	peroxidase activity (GO:0004601); heme binding (GO:0020037); hydrolase activity, hydrolyzing O-glycosyl compounds (GO:0004553)	carbohydrate metabolic process (GO:0005975); oxidation-reduction process (GO:0055114); response to oxidative stress (GO:0006979)

## Discussion

In this work, we compared the differences in detectable transcripts of nuclear genes encoding for plastid proteins in *Rafflesia*, *Phelipanche* and *Arabidopsis*. Variations in expression abundance plotted for each species reveals a relatively consistent trend of some housekeeping regulatory pathways for all species investigated, including the parasitic ones. Plastids are capable of transforming from one type to another, accompanied by the alteration of plastid proteome composition^24^. Despite some differences, both tissue and environmental signals induce changes in the plastid proteomes^24^. Therefore, a core set of essential genes is expected to be transcribed in each species. After the adoption of parasitism, relaxed selective constraints may have resulted in further plastome reduction. Additionally, the continued nonfunctionality of the plastome in parasitic plants contributes to the accumulation of mutagenic factors including microsatellites, long homopolymer tracts, forward or palindromic repeats, and a poor GC content^25^. However, these aspects of plastome evolution are not discussed here. We instead focus on how some nucleus encoded plastid genes may have been repurposed or have remained functional because their products are also core components of other non-photosynthesis pathways.

As most *ndh* and chlororespiratory genes are lost, the preferably retained ribosomal proteins and tRNAs in parasites may indicate an existence of a functional plastid DNA shift to the nuclear genomes^25^. In general, the loss of a subset of nonfunctional-related ribosomal proteins leads to a decline in plastidial translational capacity and photosynthetic performance^26^. Moreover, a complete loss of the plastidial and nuclear genes involved in the plastid DNA expression system possibly indicates the absence of a plastid genome, as proposed for the *Polytomella* genus^8^. In the case of *Rafflesia*, several plastidial ribosomal proteins along with the nucleus-encoded plastid-targeting genes were detected from the transcript data. Identification and characterization of the nucleus-encoded plastid-targeting proteins may therefore unveil the possible remaining plastidial functions. In addition, these studies provide a comparative view of the plastid genome evolution between *R*. *cantleyi* and other representatives under relaxed selective constraints.

In this study, the engagement of transit peptides and subcellular localization in the identification of nucleus-encoded plastid-targeting proteins helps to eliminate possible false positives (Fig. 2a,b), therefore, this analysis was not aimed at elucidating the true number of plastid-localized proteins. We had used the GreenCut2 inventory that was derived from protein data to identify the set of plastid-localized proteins in different species. Despite the differences between lineages, transit peptides possess, there are some similarities in amino acid composition such as the relatively high abundance of serine and threonine residues and that these residues are positively charged. This allows for transit peptide prediction through different software. We have used only the transcriptome from the flower to give an overview of the retained plastid function at the final stage of the parasites’ life cycle. Despite the true number of >2,500 plastid-localized proteins reported for flowering plants, these numbers vary greatly across species. Factors such as the sample developmental stage, pipeline used and the filtering criteria also contribute to the differences in numbers. The prediction of transit peptides and subcellular localization improved and increased the confidence level of the final output. We had also filtered the candidates through bioinformatic prediction with a cutoff ≥0.500 for TargetP analysis and this is further supplemented with manual inspection of the results. These steps help to reduce false-positives. This approach may most likely underestimate the true number of plastid-localized genes but serves to achieve the objective of identifying such genes for further analysis. We must there make clear that these results are by no means an exhaustive or complete prediction regarding the true extent of plastid localized genes. Further identification of the sequence motifs were not carried out due to the presence of multiple but non-conserved groups of nucleobases present in different transit peptides^27^.

The reconstruction of two selected pathways revealed their distinct mechanisms in *R*. *cantleyi*. The synthesis of heme is carried out by both prokaryotes and eukaryotes due to it being necessary for the function of cytochromes, chlorophylls, phycobilins, and the corrin nucleus of vitamin B_12_^28^. In non-photosynthetic organisms, heme participates in the transfer of electrons and the binding of diatomic gases^29^. The incomplete set of genes expressed in *Rafflesia* during heme biosynthesis has raised questions as to whether the plant achieves equally effective heme production. In comparison, the premature bud of the photosynthetic *Arabidopsis* seems to contain a complete set of expressed heme biosynthetic genes. It is known that even though heme biosynthesis mainly takes place in the cytoplasm and mitochondrion, synthesis of δ-amino-laevulinic acid (ALA), a key precursor, occurs in the plastid^30^. Previous studies have shown that non-photosynthetic plants retain the *trn*E gene and their associated specific transcription machinery in order to equip heme biosynthesis with mitochondrial cytochromes, P450 cytochromes and other necessary oxidative enzymes^31^. This is congruent with the proposed *trn*E hypothesis, claiming that the retention of a non-photosynthetic plastid is responsible for heme biosynthesis^30^. Heme is considered to be indispensable and is synthesized by all cells^32^. From the available data, we postulate that *Rafflesia* achieves its heme production in the plastid but discards some later stages of tetrapyrrole synthesis such as chlorophyll and perhaps, phytochromobilin formation.

Due to the differences in feeding nature, the terpenoid backbone biosynthesis between parasitic and non-parasitic plants is expected to exhibit two different mechanisms. Generally, most eukaryotes, archaebacteria, fungi, the cytosol and mitochondria of plants use the mevalonate (MVA) pathway^33^. This phenomenon is different in most eubacteria where the non-mevalonate (MEP) pathway appears to be the only pathway deployed for the biosynthesis of their isoprenoid precursors^34^. Plastid-bearing organisms, including plants, apicomplexan parasites such as *Plasmodium falciparum* (malaria) and *Toxoplasma gondii* (toxoplasmosis), also adopt the MEP pathway^35^. One intermediate product of terpenoid backbone biosynthesis produced from both the MVA and MEP pathways is IPP, and all isoprenoids are derived from this five-carbon (C_5_) compound along with its isomer dimethylallyl diphosphate (DMAPP)^34,36^. The starting substrates for both the MVA and MEP pathways are inherited from glycolysis, which are present in both photosynthetic and nonphotosynthetic plants (Fig. 4). The choice of which pathway to enter may possibly be affected by the site of synthesis; be it cytoplasmic- or plastid-located, or whether an active transport process occurs between the two organelles^37^. The synthesis of aromatic compounds in the cytoplasm suggests that the plastid in *Rafflesia* has been reduced or pseudogenized of this alternative route in providing IPP. The detection of the plastidial remnants at genome level, but not during transcriptome level analysis, has led to this interpretation. Intermediate substrates needed for sterols, sesquiterpenes and the side chain of ubiquinone biosynthesis are therefore provided by cytosolic metabolism^38^. The second hypothesis regarding this phenomenon is the lack of the above secondary metabolite biosynthesis in the parasitic *R*. *cantleyi*. The partial or selective reduction of this biosynthesis pathway can also be illustrated by the lack of detectable gene sequences participating in the carotenoid pathway. Conversely, the carotenoid pathway is present in the *Polytomella* genus but found to be at various stages of degradation in the four species studied^39^.

Ideally, the abundance of nuclear plastid DNA-like sequences (NUPTs) in nuclear genomes correlates well with the number of plastids present in an organism^40^. This phenomenon is found to be consistent across terrestrial plants, green algae, apicomplexans and stramenopiles. However, evolutionary parasitism has propelled plastid reduction and the extent of their viability depends on the degree of parasitism and plastid metabolic complexity^41^. The more diverse plastid of *Phelipanche* compared to *Rafflesia* may indicate a more recent autotrophic ancestor. Orobanchaceae was reported to have undergone massive gene loss and pseudogenisation^42^. If this was the case for Orobanchaceae, a greater gene loss in Rafflesiaceae would not be surprising. This can be attributed to the non-photosynthetic status of all *Rafflesia* species whereas some species in Orobanchaceae still retain either a complete or a collectively constrained chlorophyll synthesis pathway^11^. In contrast, non-photosynthetic tissues of photosynthetic plants are found to contain a more complete set of genes even though they do not require an active photosynthetic apparatus at this stage. Studies on the plastidial proteins encoded by the nucleus provide a rudimentary view of the possibly retained functions known to be taking place in plastids. In addition, retention of NEP-related pathways in plastid indicates an inter-connection among the actual pathways sited in the plastids to the nuclear genome encoding the genes^41^.

With the currently available technology and resources, it is highly unlikely that a large scale comparative experiment can be executed to focus solely on the question of the loss of photosynthetic capacity in diverse plants from different geographical regions. Furthermore, the cultivation and tissue culture of parasitic plants are extremely difficult thus highly dependent on the availability of field samples. However, the availability of publicly accessible genome and transcriptome data can be analysed and certain contextual insights can be extracted from them. Such approaches undoubtedly have limitations and care must also be made in extrapolating functional information from such datasets. Nevertheless, they can prove to be useful starting points for further investigations as demonstrated in this work.

## Conclusions

Comparative studies of nucleus-encoded plastid-targeting proteins of non-photosynthetic parasitic plants and the photosynthetic ones reveal the transcription status of these proteins and some prominent processes pertaining to the plastid despite plastid reduction. Fundamental roles such as amino acid biosynthesis and nucleic acid metabolism are two such examples that would seldom be discarded by any plants. The phylogeny of *Rafflesia* for two selected nucleus-encoded proteins showed no evidence that the genes encoding those functions were acquired by horizontal gene transfer, implying that they are expressed by the parasitic plant itself to fulfill its biological roles. The available evidence however suggests that the plastid genome may devolve to the nuclear genome and necessary functions are transported back to the plastid or the residual encoded plastidial functions partake in essential processes that are not related to phtosynthesis.

## Methods

### DNA sequencing and genome sequence annotation

The *Rafflesia cantleyi* flower sample was collected from Pahang, Malaysia. DNA was extracted from the perigone lobe tissue of the *Rafflesia* flower using a modified CTAB protocol^43^. Extracted genomic DNA was checked for quantity and quality using the NanoDrop 2000c machine and conventional gel electrophoresis. Ligation of specific adaptors and emulsion PCR amplification were carried out following the manufacturer’s protocol. After quality assessment, the sample was run on the Roche GS FLX Titanum sequencer. Shotgun sequencing reads generated were filtered of adaptors, and short (<50 bp) and low quality (Q_Phred_ < 20) sequences using perl scripts. Redundant reads were removed using cd-hit^44^ prior to genome sequence assembly using Newbler^45^ with default parameters (minimum overlap identity 90 bp; minimum overlap length 40 bp). All contigs and singletons were queried for gene information using BLAST v2.2.26 (http://www.ncbi.nlm.nih.gov/) (BLASTX, 1e-6)^46^ and Plant Metabolic Network (http://www.plantcyc.org/) (BLASTX, 1e-10)^47^. In addition, Blast2GO^48^ was used to annotate and classify genes according to their cellular distribution, molecular function and biological process.

### RNA sequence assembly and annotation

Total RNA of *Rafflesia* flower was isolated from the same sample using a method by López-Gomez *et al*.^49^. RNA integrity was assessed using Agilent BioAnalyzer (Agilent Technologies, USA) and the quality was examined using Spectrophotometer ND-1000 (NanoDrop, USA). After cDNA preparation and library construction using TruSeq RNA Sample Prep kit, the sample was sequenced using the Illumina Solexa platform^16^. The raw data was preprocessed and annotated using BLAST v2.2.26^46^, and Blast2GO^48^. SRA archive file for *Arabidopsis* bud (#SRR544881) tissues was downloaded from the NCBI Sequence Read Archive (http://www.ncbi.nlm.nih.gov/sra). Transcript sequences for *Phelipanche aegyptiaca* bud were downloaded from the Parasitic Plant Genome Project (http://ppgp.huck.psu.edu/download.php)^17^. All files, except for contigs downloaded for *P*. *aegyptiaca*, were trimmed for short (<50 bp) and low quality (Q_Phred_ < 20) sequences using Perl scripts. Sequences were then checked for the presence of sequencing primers using FastQC v0.7.0^50^. Clean and high quality reads were then subjected to sequence assembly using VelvetOptimiser (https://github.com/Victorian-Bioinformatics-Consortium/VelvetOptimiser)^51^ and Velvet^51^ to obtain an optimised k-mer. Oases^52^ was then used to produce flower transcripts from Velvet contigs. Assembly for the pre-processed *Arabidopsis* sequences was performed using Trinity^53^ and Oases^52^. All transcript sets were then queried using BLAST v2.2.26 (BLASTX, 1e^−5^)^46^.

### Identification of nucleus-encoded plastid-targeting proteins

For each transcriptome dataset, the annotated transcripts were compared with the GreenCut2 Inventory^13^ to produce a list of candidates for nucleus-encoded plastid-targeting proteins. The six-frame translation of the candidate plastid-localized genes was performed using EMBOSS Transeq (http://ww.ebi.ac.uk/Tools/st/emboss_transeq/). Coding sequences were predicted from the transcripts using Transdecoder^54^ on Galaxy v1.0.3.0^55^. The corresponding translated peptide sequences from EMBOSS Transeq were subjected to prediction for the presence of a plastid-targeting transit peptide using ChloroP (http://www.cbs.dtu.dk/services/ChloroP/)^56^. Initial lists of the candidate plastid-localized proteins with a predicted transit peptide were checked for their corresponding coding sequences. Candidate plastid-localized genes that lacked a predicted coding sequence were discarded from the lists. Next, the predicted peptide sequences of the candidates were used for subcellular localization prediction using TargetP v1.1 (http://www.cbs.dtu.dk/services/TargetP/)^57^ with cutoff ≥0.500. A list consisting of the final putative plastid-localized proteins was compiled from the prediction output for each dataset. The lists of putative plastid-localized proteins after ChloroP and TargetP prediction were categorized into their functional groups according to the classification system in the GreenCut2 Inventory. For two selected plastid-localized proteins, RAxML (http://embnet.vital-it.ch/raxml-bb/)^58^ was used for phylogenetic tree construction. The putative nucleus-encoded plastid-targeting proteins identified for *R*. *cantleyi* were searched for orthologous genes using OrthoMCL v5 (http://orthomcl.org/orthomcl/)^23^.

### Pathway mapping and reconstruction

Two plastid-regulating pathways commonly retained in both photosynthetic and non-photosynthetic plants were selected for *Rafflesia* pathway reconstruction. Transcript data was used for gene detection and pathway mapping. Briefly, transcript IDs in GenBank format were extracted from each blast output file and were converted to uniprotAC and KO format using an embedded tool in UniPROT (http://www/uniprot.org/)^59^. To get a first glimpse of the expressed transcripts and their respective pathways, KO IDs were mapped against KEGG reference (http://www.genome.ad.jp/kegg/) pathways. The reference pathways selected for pathway reconstruction were downloaded from KEGG (http://www.genome.ad.jp/kegg/)^60^. The detected transcripts for each regulatory step were manually inspected and included in the newly reconstructed pathways.

## Electronic supplementary material

Supplementary Figure S1

Supplementary Dataset S1

Supplementary Dataset S1
