# Supplementary material for: Comparative analysis of nucleus-encoded plastid-targeting proteins in Rafflesia cantleyi against photosynthetic and non-photosynthetic representatives reveals orthologous systems with potentially divergent functions
Source: Sci Rep. 2018 Nov 22;8:17258. doi: 10.1038/s41598-018-35173-1 (PMC6250676; doi:10.1038/s41598-018-35173-1)

Comparative analysis of nucleus-encoded plastid-targeting proteins in *Rafflesia cantleyi* against photosynthetic and non-photosynthetic representatives reveals orthologous systems with potentially divergent functions

Siuk-Mun Ng, Xin-Wei Lee, Mohd-Noor Mat-Isa, Mohd Afiq Aizat-Juhari, Jumaat Haji Adam, Rahmah Mohamed, Kiew-Lian Wan, Mohd Firdaus-Raih

Supplementary Figure S1. Phylogenetic trees of (a) riboflavin synthase and (b) lysophosphatidic acid acyltransferase, two examples of plastid-targeting proteins that are expressed in *Rafflesia cantleyi*. The tree was built using RAxML. Distant placement of the two *Rafflesia* genes with *Vitis vinifera* proposes that these genes are laterally transferred from the ancestors but not horizontally acquired from their host.

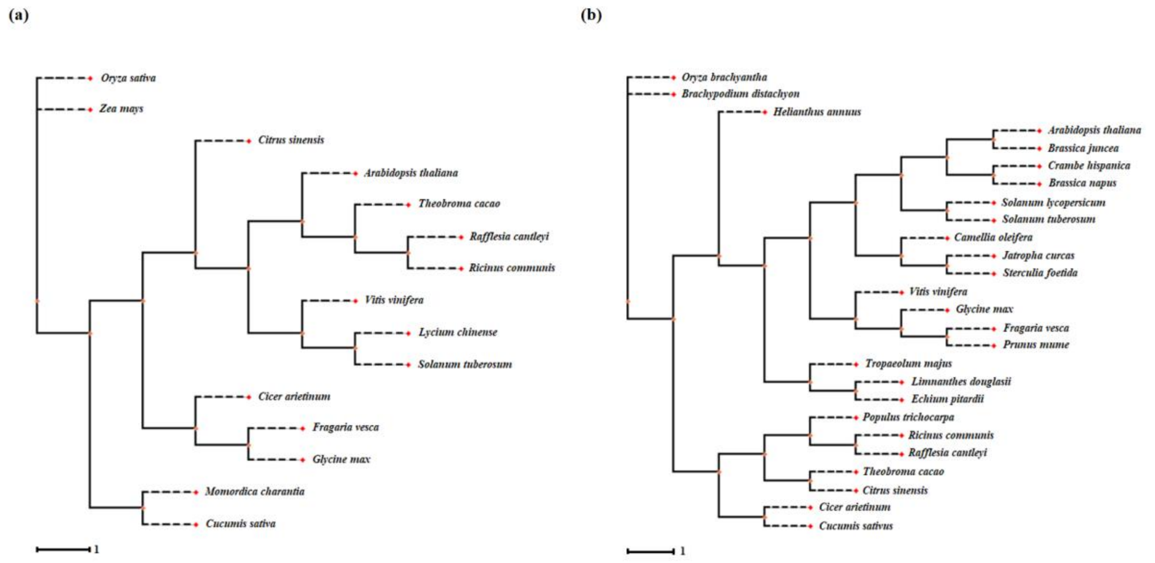

Supplement: Supplementary file 1 — Supplementary Figure S1 [file 41598_2018_35173_MOESM1_ESM.pdf]
